# Knowledge, Attitudes, and Practices Regarding Organ Donation Among Medical Students in India: A Mixed Methods Study

**DOI:** 10.7759/cureus.56136

**Published:** 2024-03-14

**Authors:** Vaibhavkumar Shrivastav, Yogesh Murugan, Rohankumar Gandhi, Jay Nagda

**Affiliations:** 1 Community Medicine, Shri M P Shah Government Medical College, Jamnagar, IND; 2 Family Medicine, Guru Gobindsingh Government Hospital, Jamnagar, IND; 3 Internal Medicine, Narendra Modi Medical College, Ahmedabad, IND

**Keywords:** india, practices, attitudes, knowledge, medical students, organ donation

## Abstract

Background: Deceased organ donation rates are extremely low in India. As future physicians, medical students can advocate organ donation in society. However, their knowledge, attitudes, and practices regarding organ donation remain understudied in India. Therefore, the present study aimed to assess the knowledge, attitudes, and practices related to deceased organ donation among undergraduate medical students in India using a mixed methods approach.

Materials and methods: This is a mixed-method study with a cross-sectional survey conducted among 400 randomly selected medical students at a medical college in India using a pretested questionnaire. Additionally, 20 in-depth interviews were conducted to gain qualitative insights.

Results: Knowledge was high regarding organ donation (90%) but lower for brain death (27.5%). Most had positive attitudes, but only 11% were registered donors, and 10% had discussed organ donation with family. Multivariate regression revealed that having third- and fourth-year-old students, urban upbringing, good knowledge, and positive attitudes were associated with increased willingness to donate. Qualitative findings revealed gaps in brain death understanding, religious myths, lack of conviction, and family disapproval as barriers.

Conclusion: Despite good awareness, gaps in the comprehension of brain death persist among students. However, the registration and family discussion rates are very low. Targeted strategies such as integrating ethical issues into medical curricula, public awareness campaigns busting myths, simplifying donor registration, and promoting family conversations are strongly recommended. This can empower students to become physician advocates driving organ donation uptake in India.

## Introduction

Organ donation involves harvesting healthy organs and tissues from deceased or living donors for transplantation into recipients suffering from end-stage organ failure [1]. Deceased donation accounts for the majority of transplants, which require determination of brain death and consent from the donor's family [2]. In recent years, the need for transplants has dramatically increased due to the increasing incidence of conditions such as diabetes, heart disease, and kidney failure [3]. However, there is a widening gap between the demand for and availability of organs globally, resulting in avoidable deaths on transplant waitlists [4].

In India, the deceased organ donation rate is very low, at 0.26 per million people [5]. Major barriers include a lack of awareness, religious myths and misconceptions, concerns about bodily disfigurement, distrust in the healthcare system, and poor family consent rates [6]. There is an urgent need to improve knowledge and attitudes to increase willingness toward deceased donations to bridge the rising demand-supply gap.

Medical students represent the future generation of physicians and can play a pivotal role in advocating organ donation in society [7]. However, studies have shown persistent myths and fears about organ donation among students worldwide [8,9]. There is limited published literature on knowledge, attitudes, and practices regarding organ donation, especially among Indian medical students.

Assessing the knowledge gaps, cultural barriers and motivators among students can help inform targeted strategies to improve their practices, which is vital for fostering positive donation behaviors among future healthcare providers. This, in turn, can enable them to educate and counsel the broader community more effectively. Hence, this study aimed to assess knowledge, attitudes, and practices regarding deceased organ donation among undergraduate medical students in India using a mixed methods approach. The findings can provide insights to guide evidence-based interventions to improve organ donation rates in the country.

## Materials and methods

Study design and setting

This mixed methods study utilized a cross-sectional quantitative survey followed by qualitative in-depth interviews. The concurrent nested strategy was used, with the qualitative component nested within the predominant quantitative approach to allow for a more comprehensive understanding of the research problem [10].

Study setting and participants

The study was conducted among undergraduate medical students at a medical college in Gujarat.

Sample size justification

The sample size was calculated using the formula of Cochran (1977) [11]:

$n=\frac{Z^{2}p(1-p)}{e^{2}}$

where n = the required sample size, Z = the z statistic for the level of confidence (1.96 for 95% confidence level), p = the expected prevalence or proportion (0.5 used for maximum variability), and e = the precision (0.05 for 5% margin of error).

Using this approach, the minimum sample size was calculated to be 384, which was rounded to 400 participants. Simple random sampling was used to select participants from each year of study at the medical college. The list of students was obtained from the institution, and random number tables were used to randomly select the required number of participants from each year. This eliminated selection bias and ensured the representativeness of the sample [12]. Additionally, 20 participants were purposively selected for in-depth interviews to gain further insight into perceptions and attitudes.

Data collection tool and technique

Quantitative Methods

A pretested, structured questionnaire adapted from previous studies [13] was self-administered to participants. Knowledge about organ donation was assessed using nine questions, attitudes were assessed with five questions, and self-reported practices were assessed with two questions.

Validity and reliability: The questionnaire was validated by experts in the field and pretested on 20 students to assess the clarity and suitability of the questions. Reliability was ensured by measuring Cronbach's alpha (>0.7).

Qualitative Methods

Individual in-depth interviews were conducted using a topic guide exploring barriers, motivators, attitudes, and beliefs regarding organ donation. The interviews were audio-recorded, transcribed verbatim, and translated for analysis. Data collection was stopped once saturation was reached.

Procedure

The questionnaire was self-administered by participants and collected by the researchers. Any doubts or clarifications needed were addressed. On average, the questionnaire took 15-20 minutes to complete.

Period: Data collection was carried out over three months from January 2023 to March 2023.

Quality assurance: Questionnaire completeness was checked daily. Any discrepancies or missing responses were clarified to the participants immediately.

Qualitative Data Collection

Participant selection: Twenty participants were purposively selected from those completing the survey to represent maximal diversity based on year of study, gender, knowledge, and attitudes regarding organ donation.

Data collection: In-depth interviews were conducted using a flexible topic guide to explore knowledge, attitudes, beliefs, motivators, and barriers regarding organ donation. The interviews lasted 30-45 minutes, were audio-recorded, and took place in a private setting.

Data saturation: Interviews were conducted iteratively until data saturation was reached and no new themes emerged.

Transcription and translation: Audio recordings were transcribed verbatim and translated into English for analysis.

Operational Definitions

Good knowledge was defined as answering ≥50% of the knowledge questions correctly. A positive attitude was defined as having an answer to ≥50% of the attitude statements correctly. Willingness to donate was defined as a positive response to the question of willingness to donate organs after death.

Data Analysis

Quantitative data were analyzed using descriptive statistics and tests of significance, such as chi-square tests and multivariate logistic regression, with SPSS version 20 (IBM Corp., Armonk, NY). Qualitative data were coded and categorized into themes using thematic analysis [14]. Quotes illustrating common themes were identified.

## Results

Among the 400 participants, the majority were aged 18-20 years (224, 56%), and there was an equal distribution of males (192, 48%) and females (208, 52%). Most were in their 1st (140, 35%) or 2nd (128, 32%) year of study. More participants were from urban areas (242, 60.5%) than from rural areas (158, 39.5%). The predominant religion was Hindu (310, 77.5%), followed by Islam (50, 12.5%) and other religions (40, 10%). Close to half had fathers with primary/secondary education (184, 46%) (Table 1).

**Table 1:** Sociodemographic characteristics of the participants

Sociodemographic Characteristic	Frequency (n=400)
Age	
18-20 years	224 (56%)
21-23 years	120 (30%)
≥24 years	56 (14%)
Gender	
Male	192 (48%)
Female	208 (52%)
Year of Study	
1st year	140 (35%)
2nd year	128 (32%)
3rd year	80 (20%)
4th year	52 (13%)
Place of Upbringing	
Urban	242 (60.5%)
Rural	158 (39.5%)
Religion	
Hindu	310 (77.5%)
Islam	50 (12.5%)
Other	40 (10%)
Father's Education	
Below Primary	32 (8%)
Primary/Secondary	184 (46%)
Higher Secondary	86 (21.5%)
Graduate/Postgraduate	98 (24.5%)

Knowledge was high for awareness about organ donation (90%) and organs that can be donated (83.6%) but lower for understanding brain death (27.5%). Most had positive attitudes, such as agreeing that religion allows organ donation (76.5%) and that it should be promoted (91%). However, only a small proportion were registered organ donors (44, 11%) or had registered friends/family (40, 10%) (Table 2).

**Table 2:** Knowledge, attitudes, and practices of the participants regarding organ donation

Domain	Questions	Aware/Correct n (%)
Knowledge		
	Awareness about organ donation	360 (90)
	Knowledge of organs donated	334 (83.6)
	Awareness of donor registry	238 (59.5)
	Understanding brain death	110 (27.5)
	Awareness of live donation	148 (37)
Attitudes		
	Religion allows organ donation	306 (76.5)
	Should be promoted	364 (91)
	Registration promotes donation	368 (92)
	More information needed	252 (63)
	Want donor registration info	160 (40)
Practices		
	Registered organ donors	44 (11)
	Friends/family registered donors	40 (10)

The prevalence of knowledge was greater among those aged ≥24 years (40, 71%), those aged 18-20 years (124, 55%, p>0.05), and urban residents (150, 62%) than among rural residents (74, 47%, p<0.01). Females (134, 64%) had better knowledge than males (90, 47%, p<0.01).

Positive attitudes did not significantly differ. The willingness to donate organs was greater among those in their third year (52, 65%) than among those in their first year (44, 31%, p<0.05) and among urban residents (124, 51%) than among rural residents (60, 38%, p<0.05) (Table 3).

**Table 3:** Association between sociodemographic characteristics and knowledge, attitudes, and practices of the participants regarding organ donation *p<0.05 - significant, ** p<0.01 – highly significant

Sociodemographic Factor	Good Knowledge (n=224)	Positive Attitude (n=340)	Willingness to Donate Organs (n=184)
Age			
18-20 years	124 (55%)	196 (88%)	104 (46%)
21-23 years	60 (50%)	96 (80%)	56 (47%)
≥24 years	40 (71%)	48 (86%)	24 (43%)
Year of Study			
1st year	64 (46%)	112 (80%)	44 (31%)
2nd year	56 (44%)	116 (91%)	60 (47%)
3rd year	52 (65%)*	64 (80%)	52 (65%)*
4th year	52 (100%)**	48 (92%)	28 (54%)
Gender			
Male	90 (47%)	172 (89%)	96 (50%)
Female	134 (64%)**	168 (88%)	88 (42%)
Place of Upbringing			
Urban	150 (62%)**	216 (89%)	124 (51%)
Rural	74 (47%)	124 (78%)	60 (38%)*

The willingness to donate organs was significantly greater among those in their third year (OR 3.92, p<0.001) and fourth year (OR 2.64, p=0.02) than among those in their first year. Good knowledge (OR 1.86, p=0.02) and positive attitude (OR 3.45, p<0.001) were also associated with increased willingness (Table 4).

**Table 4:** Multivariate logistic regression for factors associated with willingness to donate organs *p<0.05 - significant, ** p<0.01 – highly significant

Variable	Crude OR (95% CI)	Adjusted OR (95% CI)	P-value
Age			
18-20 years	Ref	Ref	
21-23 years	1.00 (0.59-1.71)	1.01 (0.59-1.72)	0.98
≥24 years	0.89 (0.38-2.05)	0.87 (0.37-2.01)	0.74
Year of Study			
1st year	Ref	Ref	
2nd year	1.97 (1.00-3.89)	1.94 (0.98-3.84)	0.06
3rd year	4.01 (1.91-8.42)	3.92 (1.87-8.24)	<0.001 **
4th year	2.69 (1.18-6.17)	2.64 (1.15-6.05)	0.02 *
Gender			
Male	Ref	Ref	
Female	0.70 (0.43-1.16)	0.71 (0.43-1.18)	0.19
Place of Upbringing			
Rural	Ref	Ref	
Urban	1.71 (0.96-3.03)	1.69 (0.95-3.00)	0.07
Religion			
Hindu	Ref	Ref	
Muslim	0.70 (0.29-1.72)	0.71 (0.29-1.75)	0.45
Other	0.57 (0.21-1.53)	0.58 (0.21-1.57)	0.29
Good Knowledge	1.85 (1.12-3.05)	1.86 (1.12-3.08)	0.02 *
Positive Attitude	3.51 (1.70-7.27)	3.45 (1.67-7.12)	<0.001 **

Key barriers were knowledge gaps about brain death and organ donation processes, religious beliefs regarding the sanctity of the body and afterlife, fears about surgery and funeral delays, lack of exposure and awareness, apathy and laziness, mistrust in the system, and family disapproval (Table 5).

**Table 5:** Qualitative themes on barriers to organ donation

Theme	Subtheme	Quote
Knowledge gaps	Brain death concept	"I'm not 100% sure how brain death is defined or when organs can be donated."
	Organ donation process	"The actual donation process seems confusing to me."
Religious beliefs	Sanctity of human body	"I have been taught not to desecrate the body as it is made by God."
	Afterlife concerns	"What if I need all my organs in the next life?"
Fear	Surgery	"I'm afraid of how donating organs would affect my body."
	Funeral delays	"I worry organ donation disrupts funeral rites and ceremonies."
Lack of awareness	No exposure	"I never learned or talked about organ donation growing up."
	Not a priority	"I do not actively think about organ donation as an issue."
Apathy	Laziness	"I cannot be bothered to register as an organ donor."
	Lack of conviction	"Organ donation appears to be a good thing but it is not important to me."
Mistrust in system	Black market	"There could be organ trade rather than legal donation."
	Misuse of organs	"I worry my organs may not be allocated properly."
Family disapproval	Against wishes	"My family would not allow donating my organs after death."
	Emotional barrier	"It would be upsetting for my family if I donated organs."

## Discussion

This mixed methods study revealed good awareness but gaps in knowledge about organ donation among medical students, highly positive attitudes, and low rates of donor registration and family discussions.

Knowledge

In our study, 90% of the students were aware of organ donation. However, only 27.5% of the respondents understood brain death accurately, comparable to the 30.5% reported in an Indian study [13]. However, in Saudi studies, men (13.07%) and women (13.60%) [14] were more likely to know about organ donation. This highlights persisting gaps in the comprehension of brain death despite generally high awareness.

Attitudes

A large majority (91%) felt that organ donation should be promoted and was consistent with their beliefs. These findings are greater than those of Ethiopia and Spain, who reported values of 62.8% and 80%, respectively [15,16]. This figure is also greater than that of other Indian studies conducted in Karnataka, which reported a value of 71.3% [17]. More refuting common myths and addressing misconceptions through medical education could further strengthen positive outlooks.

Practices

Only 11% were registered organ donors. Family discussion rates (10%) were also low and concerning, as families can override donor wishes. When comparing these findings with those of previous studies, according to a study in the United States, while the majority of Americans supported organ donation (95%), only 58% [18] were registered donors, and another Indian study in Guwahati reported that 3.33% of participants signed up for organ donation [19]. Improving registration and family communication should be priorities.

Influencing factors

Multivariate analysis revealed that senior education, urban upbringing, good knowledge, and positive attitudes were associated with increased willingness to donate organs, which is consistent with previous findings [20]. The qualitative findings also highlighted knowledge gaps, religious myths, lack of exposure, and family disapproval as key barriers. A study in the United States revealed that common misconceptions about organ donation, including religious myths, were barriers to donation [21]. A focus group study in Australia revealed that lack of exposure and knowledge about the organ donation process, as well as religious and cultural beliefs, were barriers to organ donation. Another focus group study in Australia revealed that perceived religious prohibition, cultural myths and misperceptions, and distrust of the medical system were barriers to organ donation [22]. A qualitative study in China revealed that traditional beliefs, lack of knowledge, and mistrust in the donation process were barriers to organ donation [23]. A thesis on barriers to organ donation revealed that lack of knowledge, religious beliefs, and family disapproval were common barriers to organ donation [24].

Recommendations

Several strategies can help improve knowledge, attitudes, and organ donation practices among medical students. The medical curriculum should incorporate comprehensive education on brain death determination, donation processes, and ethical-legal aspects through lectures, small group discussions, and role-playing [25]. Culturally appropriate awareness campaigns utilizing print, digital media, street plays, and testimonials can address myths and misconceptions about organ donation in the community [26]. Simplifying organ donor registration by allowing online applications and through college drives can improve donor registration rates. Workshops facilitating discussions about organ donation wishes with family members may empower students to have these conversations. Peer champion programs involving senior donor-registered students promoting organ donation could leverage peer influence. Multisectoral efforts between universities, policymakers, and healthcare systems are needed to execute impactful strategies.

Limitations

This study has certain limitations, including being conducted at a single medical college and relying on self-reported practices. The knowledge questionnaire also did not use vignettes to assess applied understanding. The qualitative component had a small purposive sample.

## Conclusions

This study provides valuable insights into the knowledge, attitudes, and practices regarding organ donation among Indian medical students. Although awareness is high, there are persisting knowledge gaps and poor registration/family discussion rates. Focused strategies such as integrating ethical issues into medical teaching, cultural-centric public education, simplifying registration procedures, and promoting family conversations are recommended to improve donation practices. The wide implementation of evidence-based interventions can help medical students emerge as future physicians advocate inspiring broader organ donation uptake in the community.
